# Effects of IMOD™ and Angipars™ on mouse *D*-galactose-induced model of aging

**DOI:** 10.1186/2008-2231-20-68

**Published:** 2012-10-27

**Authors:** Samane Ghanbari, Mahsa Yonessi, Azadeh Mohammadirad, Mahdi Gholami, Maryam Baeeri, Hamid Reza Khorram-Khorshid, Farhad Gharibdoost, Mohammad Abdollahi

**Affiliations:** 1 Pharmaceutical Sciences Branch, Islamic Azad University, Tehran, 1941933111, Iran; 2 Department of Toxicology and Pharmacology, Faculty of Pharmacy, and Pharmaceutical Sciences Research Center, Tehran University of Medical Sciences (TUMS), Tehran, 1417614411, Iran; 3 Genetic Research Center, University of Social Welfare and Rehabilitation Science, Tehran, 1981619573, Iran; 4 Rheumatology Research Center, TUMS, Tehran, 141556157, Iran

**Keywords:** Aging, *D*-galactose, Oxidative stress, Cytokines, IMOD, Angipars, Animal

## Abstract

The aim of this study was to evaluate the effects of two registered herbal drugs called IMOD and Angipars on mouse model. Aging was induced by *D*-galactose (500 mg/kg) administered to animals for 6 weeks through drinking water. Male BALB/c mice were randomly divided into 5 groups receiving *D*-galactose (*D*-galactose, 500 mg/kg) for 6 weeks; positive control (*D*-galactose [500 mg/kg] for 6 weeks + Vitamin E [200 mg/kg/day] intraperitoneally for 4 weeks); IMOD (*D*-galactose [500 mg/kg] for 6 weeks + IMOD [20 mg/kg/day] intraperitoneally for 4 weeks), Angipars (*D*-galactose [500 mg/kg] for 6 weeks + Angipars [2.1 mg/kg/day] by gavage for 4 weeks); and the fifth group that was sham and not given *D*-galactose. At the end of treatment, pro-inflammatory markers including tumor necrosis factor-α (TNF-α), interlukine-1β (IL-β), interlukine-6 (IL-6), Nuclear Factor-kappaB (NF-κb), total antioxidant power (TAP), lipid peroxides (LPO) and male sex hormones i.e. testosterone and dehydroepiandrosterone-sulfate (DHEA-S) were measured in the blood.

Results showed that *D*-Galactose induces a significant oxidative stress and proinflammatory cascade of aging while both IMOD and Angipars recovered all of them. Interestingly, IMOD and Angipars were better than Vitamin E in improving male sex hormones in aged mice. This effect is so important and should be considered as an advantage although it cannot be explained with current knowledge. The conclusion is that IMOD and Angipars have marked anti-aging effect on *D*-galactose-induced model of aging.

## Introduction

Use of natural medicines was increased in the world because of their lower adverse effects, price and good efficacy in most human illnesses [1-3]. Recent experimental and clinical studies have confirmed anti-aging effects of some traditionally-used herbs [4]. In the recent years, the novel registered drug named (IMOD) has shown positive effects on reduction of oxidative stress and pro-inflammatory status in various studies [5]. IMOD is a combination of ethanolic extracts of *Rosa canina*, *Tanacetum vulgare* and *Urtica dioica* that are combined with selenium and urea and then exposed to a pulsed electromagnetic field [6]. Angipars is also a registered drug derived from a plant named *Melilotus officinalis* under electromagnetic processes which has marked antioxidant effects and is used as a drug of choice for management of human diabetic foot [7,8]. Both IMOD and Angipars have been approved by Iranian Food and Drug Organisation for their main effects including human immunodeficiency syndrome and diabetic foot, respectively.

Aging as an extremely complex biological phenomenon is demonstrated by accumulation of deleterious changes during the time with an increase in the chance of disease and death. The free radical and oxidative stress theory of aging is recognized as one of the most plausible and promising explanations for the process of aging [9]. Aging is associated with dysregulated immune system, weakened sex hormones and increased amount of oxidative stress markers or inflammatory cytokines.

In the present study, a typical mouse *D*-galactose-induced model of aging with some modifications was established. *D*-galactose is a reducing sugar that at higher levels is converted to aldose and hydroperoxide under the catalysis of galactose oxidase, resulting in the generation of a superoxide anion and oxygen-derived free radicals [10]. These changes are considerably similar to the normal aging process and similar to natural senescence model demonstrating neurological impairment, decreased activity of antioxidant enzymes, and poor immune responses [11,12].

Thus, given the knowledge of the antioxidant and anti-inflammatory potential of IMOD and Angipars, and the pathophysiology of aging, the present study was designed to evaluate the effects of these compounds, on *D*-galactose-induced aged mice.

## Materials and methods

### Chemicals

Thiobarbituric acid (TBA), trichloroacetic acid (TCA), n-butanol, hexadecyltrimethyl ammonium bromide (HETAB), tri (2-pyridyl)-s-triazine (TPTZ), HCl, malondialdehyde (MDA), ferric chloride (FeCL3-6H2O), D-galactose, and vitamin E (Trolox) were purchased from Merck chemical Co. (Germany). Rat specific tumor necrosis factor-α (TNF-α), interlukine-1β (IL-β), interlukine-6 (IL-6), Nuclear Factor-kappaB (NF-κb) ELISA kits were purchased from BenderMed Systems Inc. (Austria). Testosterone and dehydroepiandrosterone ELISA kits were purchased from Dia Metra (Italy). IMOD and Angipars were obtained from Parsrus Research Group (Iran).

### Animals

Male BALB/c mice (3 months old, 18–22 g) were provided from Tehran University of Medical Sciences (TUMS) animal house. The animals were housed in standard polypropylene cages with wired-net top in a controlled room (temperature 23 ± 1°C, humidity 55 ± 10%, 12-h light–dark cycle and were allowed free access to standard laboratory pellet diet and water during the experiments. All ethical issues on the use of animals were carefully considered and the study protocol was approved by TUMS review board with code number of 90-03-33-15668.

### Experimental design

Before starting the main study, a pilot was designed to set up aging model and to get proper doses of treatments. In the main study, fifty mice were randomly divided into five groups, each consisting of 10 animals. *D*-galactose was dissolved in a measured quantity of mice drinking water. *D*-galactose was given to four out of five groups of animals at 500 mg/kg *D*-galactose per 10 ml drinking water for 6 weeks [13,14]. The fifth group of animals was the sham group which was not given *D*-galactose. After 2 weeks, the four groups which had been given *D*-galactose were randomly divided into aging control group (500 mg/kg *D*-galactose per 10 ml drinking water, for 4 weeks), positive control group (500 mg/kg *D*-galactose per 10 ml drinking water plus vitamin E 200 mg/kg/day intraperitoneally for 4 weeks) and IMOD treatment group (500 mg/kg *D*-galactose per 10 ml drinking water plus IMOD 20 mg/kg/day intraperitoneally for 4 weeks), and Angipars treatment group (500 mg/kg *D*-galactose per 10 ml drinking water plus Angipars 2.1 mg/kg/day by gavage for 4 weeks).

Twenty-four hours after the last drug administration, blood samples were taken of each animal under anesthesia by cardiac puncture. Serum samples were obtained by centrifuging the whole blood at 1000 × g at 4°C for 10 minutes and the supernatants were transferred into several microtubes for separate biochemical assays and maintained at −80°C until the analyses were performed. Biochemical markers including TNF-α, IL-β, IL-6, NF-κb, ferric reducing/total antioxidant power (TAP), lipid peroxidation (LPO) marker and male sex hormones including testosterone and dehydroepiandrosterone-sulfate (DHEA-S) were measured in the serum.

### Measurement of LPO in serum of *D*-galactose-induced aged mice

LPO was measured by the reaction of TBA with MDA and other lipid peroxides. Samples were mixed with TCA (20%) and the precipitate was dispersed in H2SO4 (0.05 M). After addition of TBA (0.2% in sodium sulfate), the sample was heated for 30 min in a boiling water bath. Then LPO adducts were extracted by n-butanol and absorbance was measured at 532 nm as described in details in our previous work [15]. Data were expressed as nM.

### Measurement of TNF-α, IL-1β, IL-6 and NF-κb levels in serum of *D*-galactose-induced aged mice

Quantitative detection of TNF-α, IL-1β, IL-6 and NF-κb levels in serum were performed using an enzyme-linked immunosorbaent assay rat specific ELISA kit. The absorbance of the final colored product was measured in 450 nm as the primary wave length and 620 nm as the reference wave length. TNF-α, IL-1β, IL-6 and NF-κb levels were expressed as pg/mg.

### Measurement of TAP in the serum of *D*-galactose-induced aging mice

Serum TAP was evaluated by measuring the ability to reduce Fe^3+^ to Fe^2+^. Interaction of TPTZ with Fe^2+^ results in formation of a blue color with a maximum absorbance at 593. The whole procedure has been described in our previous study [16]. Data were expressed as mM.

### Measurement of testosterone and DHEA-S in the serum of *D*-galactose-induced aged mice

For determination of testosterone and DHEA-S, we used ELISA kits and did as instructed by the kit brochure. Testosterone and DHEA-S levels were expressed as ng/ml.

### Statistical analysis

Results are expressed as mean ± standard error of the mean (SEM). Data were analyzed by one-way ANOVA followed by Tukey post-hoc test for multiple comparisons to ensure the variances of the data are distributed properly. A p-value less than 0.05 was considered significant.

## Results

### Effects of IMOD and Angipars on LPO of aged mice

**Figure 1:**
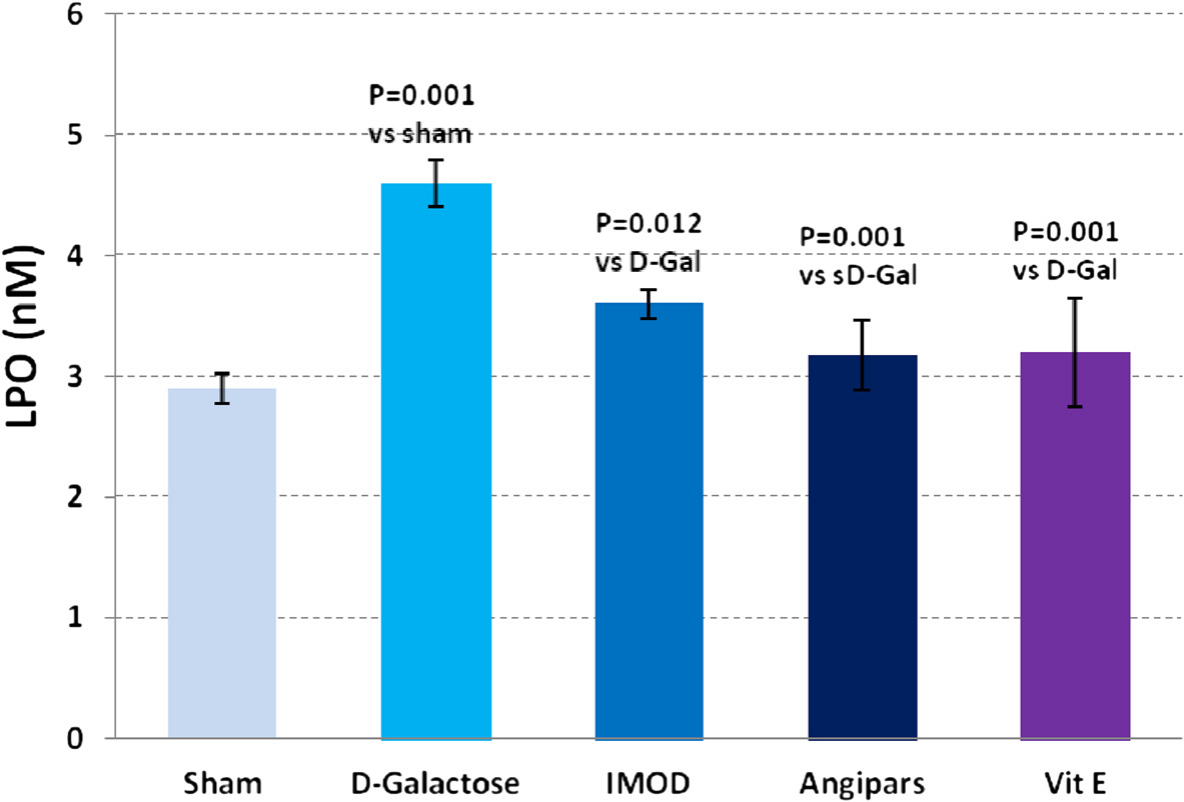
**Effects of IMOD and Angipars in serum lipid peroxidation (LPO) of *D*-galactose (D-Gal)-induced aged mice.** Data are mean ± SEM of ten animals.

The level of changes in LPO in different studies is demonstrated in Figure 1. A significant increase in LPO is evident in aged animals when compared to sham (4.6 ± 0.19 vs. 2.9 ± 0.12, P = 0.001). *D*-galactose-induced elevation of LPO was significantly restored following treatment with IMOD (3.6 ± 0.12 vs. 4.6 ± 0.19, P = 0.012), Angipars (3.17 ± 0.29 vs. 4.6 ± 0.19, P = 0.001), and vitamin E (3.2 ± 0.45 vs. 4.6 ± 0.19, P = 0.001).

### Effects of IMOD and Angipars on TAP of aged mice

**Figure 2:**
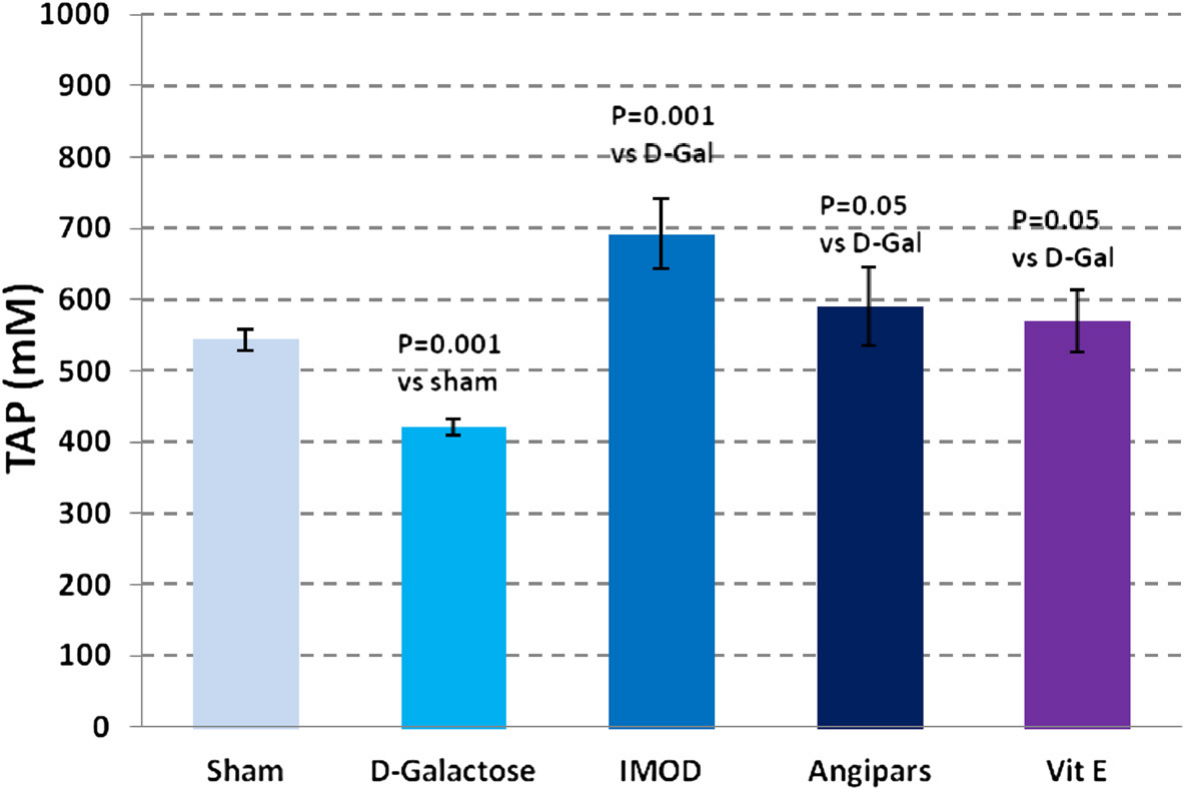
**Effects of IMOD and Angipars in serum total antioxidant power (TAP) of *D*-galactose (D-Gal)-induced aged mice.** Data are mean ± SEM of ten animals.

As shown in Figure 2, TAP in aged animals is lower than that in the sham group (544 ± 14.8 vs. 421 ± 11.3, P = 0.001). *D*-galactose-induced reduction of TAP was significantly recovered following treatment with IMOD (692 ± 49.2 vs. 421 ± 11.3, P = 0.001), Angipars (590 ± 54.9 vs. 421 ± 11.3, P = 0.05), and vitamin E (570 ± 44 vs. 421 ± 11.3, P = 0.05).

### Effects of IMOD and Angipars on TNF-α, IL-6, IL-1β and NF-kB of aged mice

**Figure 3:**
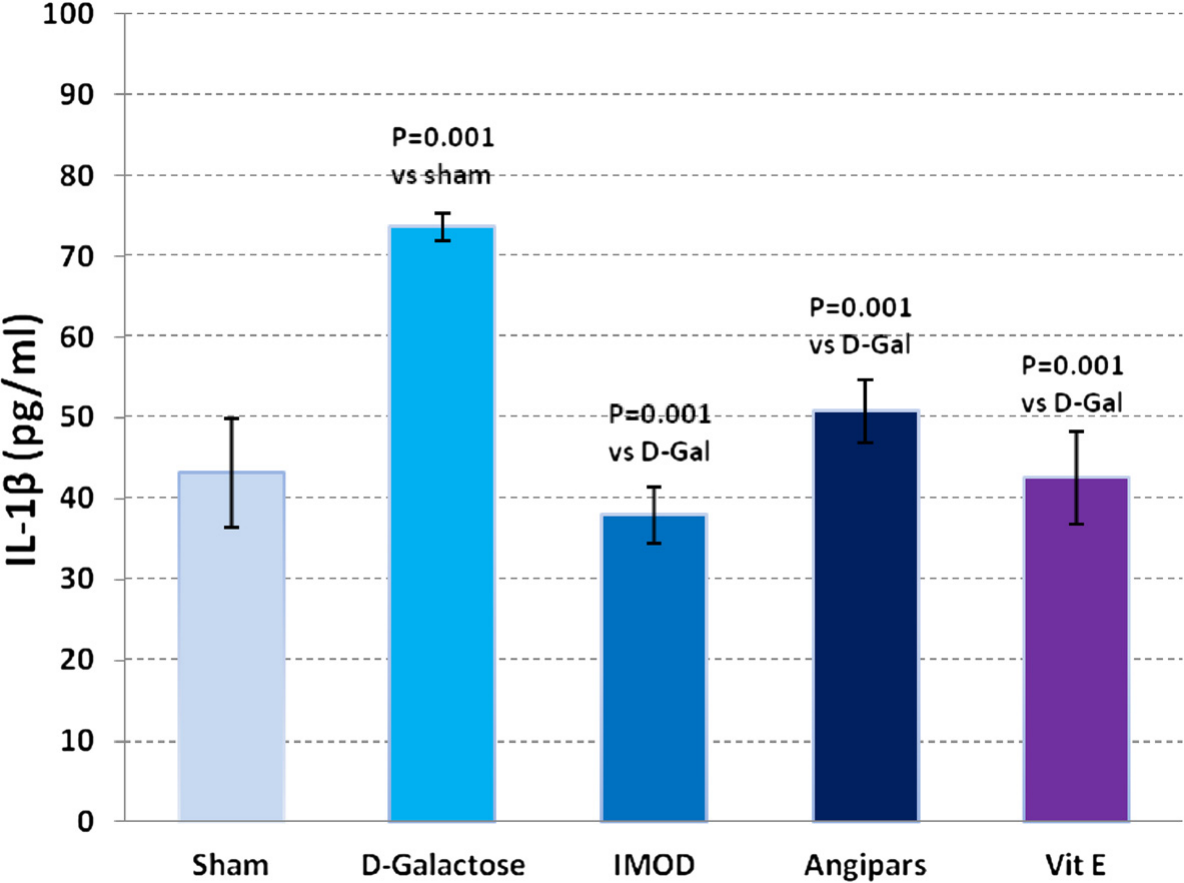
**Effects of IMOD and Angipars in serum interleukin-1 beta (IL-1β) of *D*-galactose (D-Gal)-induced aged mice.** Data are mean ± SEM of ten animals.

**Figure 4:**
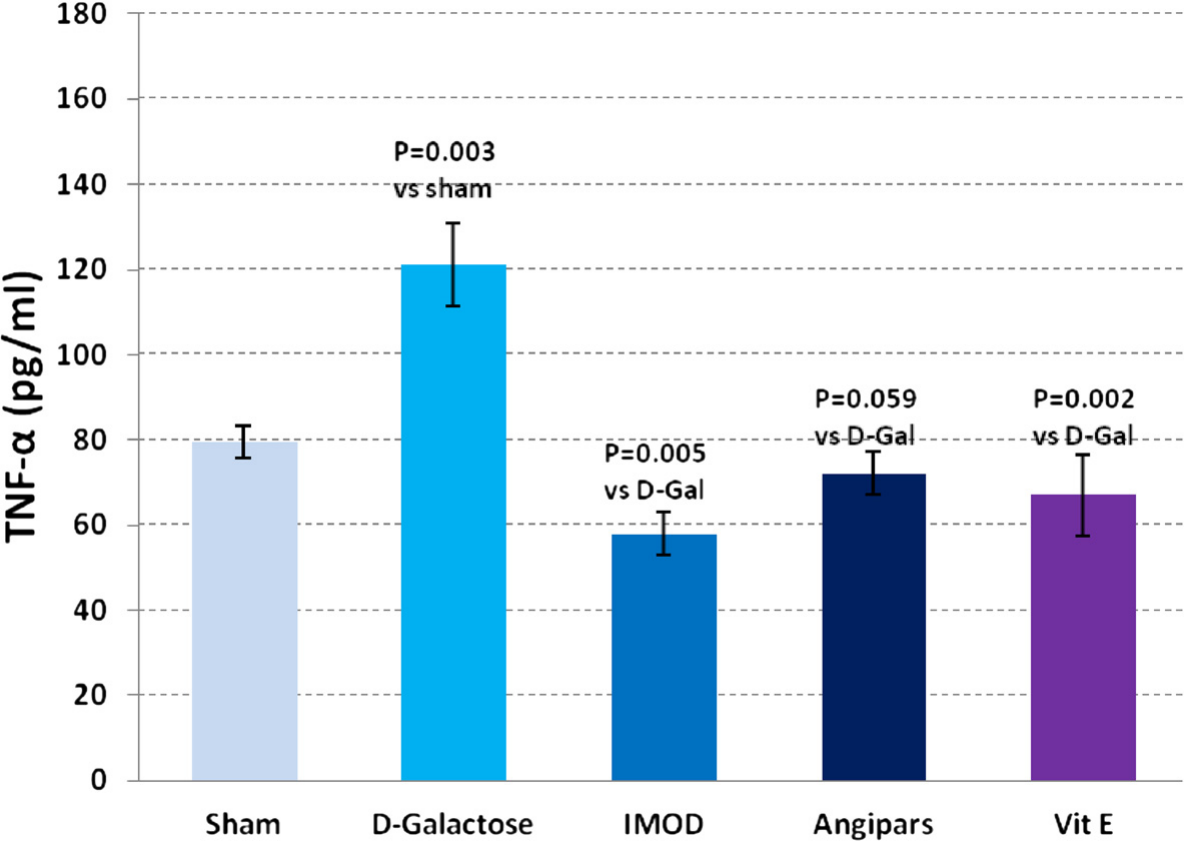
**Effects of IMOD and Angipars in serum tumor necrosis factor-alpha (TNF-α) of *D*-galactose (D-Gal)-induced aged mice.** Data are mean ± SEM of ten animals.

**Figure 5:**
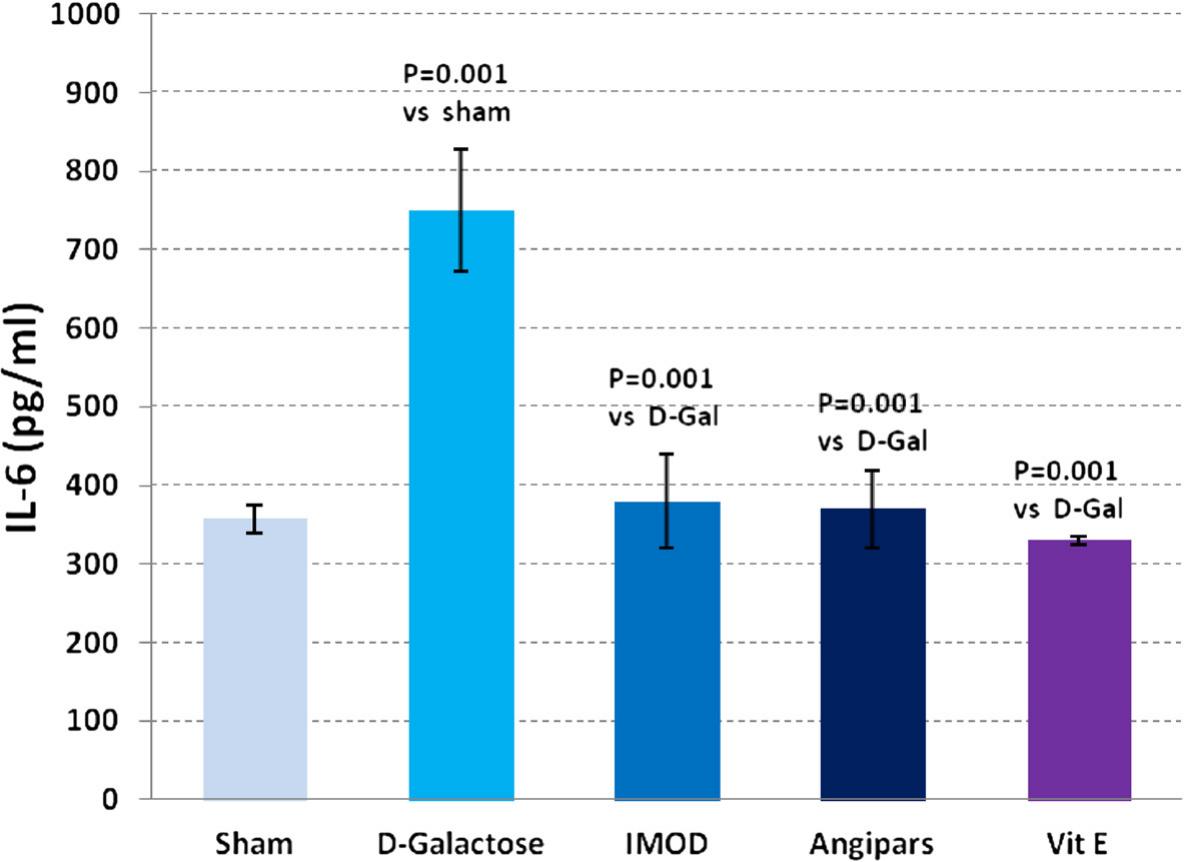
**Effects of IMOD and Angipars in serum interleukin 6 (IL-6) of *D*-galactose (D-Gal)-induced aged mice.** Data are mean ± SEM of ten animals.

**Figure 6:**
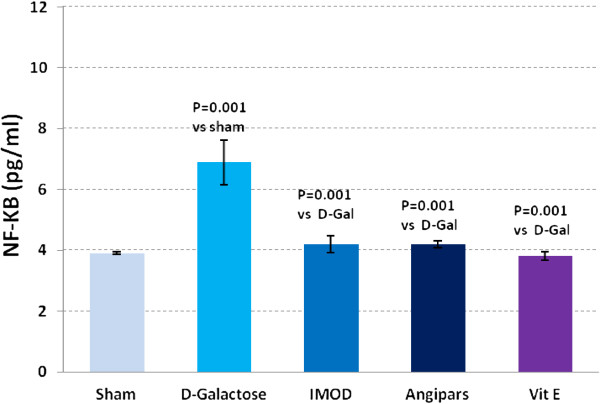
**Effects of IMOD and Angipars in serum NF-kappaB (NF-kB) of *D*-galactose (D-Gal)-induced aged mice.** Data are mean ± SEM of ten animals.

Figures 3, 4, 5 and 6 respectively show the effects of aging on the levels of TNF-α, IL-6, IL-1β, and NF-kB in comparison to sham group (121 ± 9.8 vs. 79.6 ± 3.67, P = 0.003; 750 ± 77 vs. 358 ± 17.85, P = 0.001; 73.6 ± 1.7 vs. 43.18 ± 6.7, P = 0.001; 6.9 ± 0.73 vs. 3.9 ± 0.04, P = 0.001). Administration of IMOD (58 ± 5.2 vs. 121 ± 9.8, P = 0.005; 480 ± 59.8 vs. 750 ± 77, P = 0.001; 38 ± 3.5 vs. 73.6 ± 1.7, P = 0.001; 4.2 ± 0.27 vs. 6.9 ± 0.73, P = 0.001), Angipars (72.1 ± 5.02 vs. 121 ± 9.8, P = 0.059; 477 ± 50 vs. 750 ± 77, P = 0.001; 50.8 ± 3.9 vs. 73.6 ± 1.7, P = 0.001; 4.2 ± 0.12 vs. 6.9 ± 0.73, P = 0.001) and Vitamin E (67 ± 9.4 vs. 121 ± 9.8, P = 0.002; 360 ± 5.9 vs. 750 ± 77, P = 0.001; 42.6 ± 5.7 vs. 73.6 ± 6.7, P = 0.001; 3.8 ± 0.14 vs. 6.9 ± 0.73, P = 0.001), respectively, recovered *D*-galactose-induced increase in TNF-α, IL-6, IL-1β and NF-kB of aged mice.

### Effects of IMOD and Angipars on sex hormone levels of aged mice

**Figure 7:**
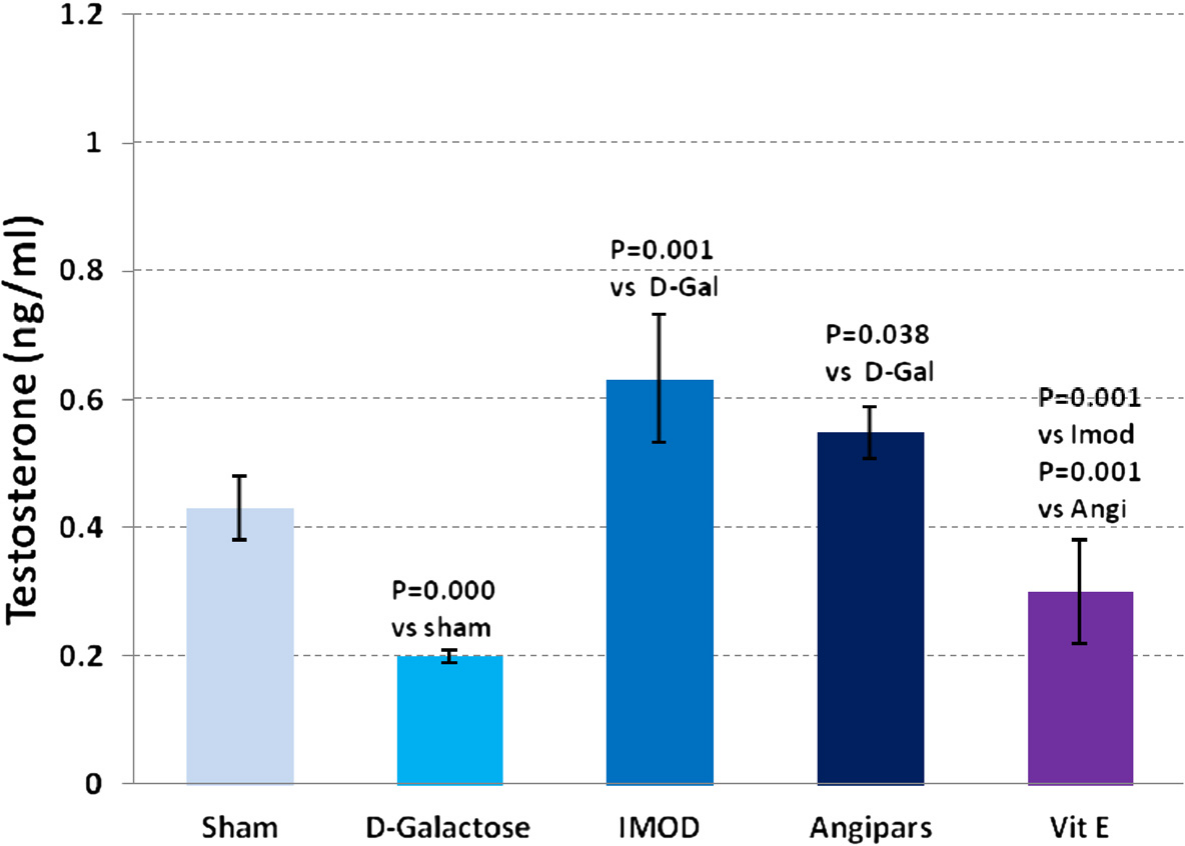
**Changes in serum testosterone of *D*-galactose (D-Gal)-induced aged mice.** Data are mean ± SEM of ten animals.

**Figure 8:**
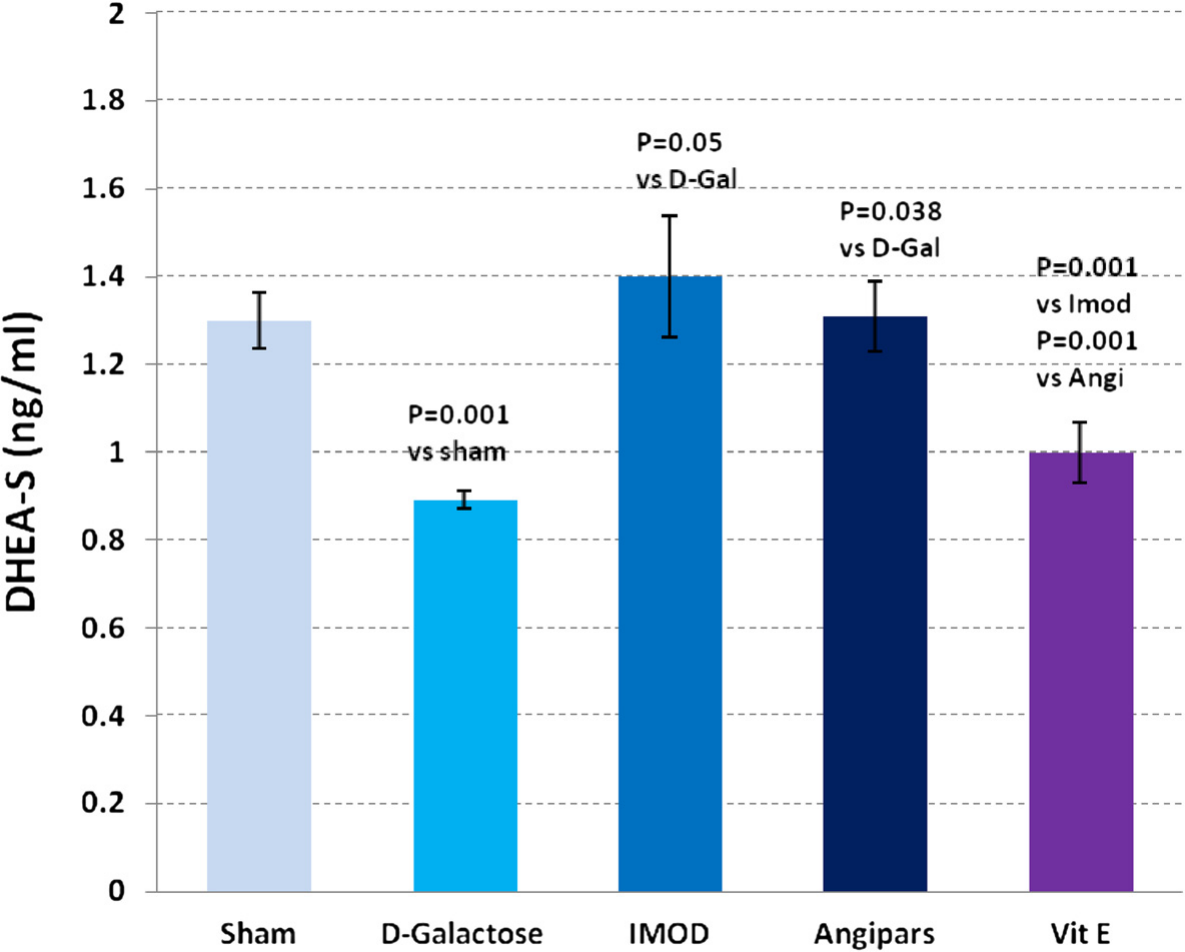
**Changes in serum dehydroepiandrosterone-sulfate of *D*-galactose (D-Gal)-induced aged mice.** Data are mean ± SEM of ten animals.

Testosterone (Figure 7) in aged mice was lower than that in the sham group (0.25 ± 0.01 vs. 0.43 ± 0.05, P = 0.000). IMOD and Angipars increased testosterone, respectively (0.63 ± 0.1 vs. 0.25 ± 0.01, P = 0.001), and (0.55 ± 0.04 vs. 0.25 ± 0.01, P = 0.038). DHEA-S (Figure 8) in aged mice was lower than that in the sham group (0.89 ± 0.02 vs. 1.3 ± 0.06, P = 0.001). IMOD and Angipars recovered both testosterone and DHEA-S in aged mice (1.4 ± 0.14 vs. 0.89 ± 0.02, P = 0.05), and (1.31 ± 0.08 vs. 0.89 ± 0.02, P = 0.034), respectively. Vitamin E could not significantly recover testosterone and DHEA-S values in aged mice when compared to aged mice (0.37 ± 0.08 vs. 0.25 ± 0.01, P = 0.09) and (1 ± 0.07 vs. 0.89 ± 0.02, P = 0.1), respectively.

## Discussion

In the present study, we showed for the first time the anti-aging potentials of IMOD and Angipars. Furthermore, these drugs dramatically improved oxidative stress status and proinflammatory cytokines of aged mice. Also, the present study confirmed that generation of free radicals that causes up-regulation of pro-inflammatory cytokines is a major determinant involved in the *D*-galactose-induced aging model. We explored that IMOD and Angipars exert their effects mainly through strong antioxidative potential and enhancing TAP and reduction of LPO. Interestingly, present results indicated improvement of testosterone and DHEA-S by IMOD and Angipars in the aged mice. Decline of steroid hormones with aging is already known and is believed a major contributor to elevation of pro-inflammatory markers [16,17]. Supporting the mechanism of action of IMOD and Angipars, and the theory of oxidative stress in aging, vitamin E showed the similar effects in examined markers of aging.

The herbs used in the IMOD complex have strong anti-oxidative stress potential that is useful in many oxidant-related diseases [1]. IMOD with its mechanism of action has been found useful in oxidative-stress-related disorders and immunoinflammatory-based diseases like type 1 diabetes [18], colitis [19], pancreatic Langerhans islet transplant [20] and polycystic ovary syndrome [21]. In addition, IMOD has been found safe in preclinical [22] and clinical studies [23] that favors its advantages if supplemented chronically to those getting old.

On the other hand, Angipars has been studied in all steps of clinical trial and was recently presented as a novel treatment for diabetic foot ulcers, with a possible mechanism of angiogenesis [24-26]. In aging the process of angiogenesis is disturbed and thus Angipars might act through increasing angiopoietins that are essential contributions to the maturation, stabilization, and remodeling of the vasculature. Angiogenesis is one of the mechanisms that responsible in the wound healing process and is declined in aging [27,28].

Interestingly, IMOD and Angipars were better than vitamin E in improving male sex hormones that are declined in aged mice. This effect is so important and should be considered as an advantage although it cannot be explained with current knowledge. This means that IMOD and Angipars are possibly acting with mechanisms rather than antioxidant effects that remain to be elucidated in further studies. Meanwhile, the combination of IMOD and Angipars should be trialed in clinic.

## Conclusion

The findings signify the potential of IMOD and Angipars as a promising source of natural antioxidants in aging.

## Competing interests

The authors declare that they have no competing interests. H.R. Khorram-Khorshid and Farhad Gharibdoost are advisers to ParsRus Research Group as developer of IMOD and Angipars. Since Mohammad Abdollahi is Editor-in-Chief of DARU, all review process of the submission was handled by one of Section Editors.

## Authors’ contributions

SG and MY carried out experimental parts of the study. AM contributed in design of the study, statistical analysis, and drafting the manuscript. MG helped in animal part of the study. MB helped in analyses of biochemical markers. HRKK and FG prepared drugs and were consulted during study. MA gave the idea, designed the study, supervised whole parts, and edited the paper. All authors read and approved the final manuscript.
